# PReS-FINAL-2263: Progressive pseudorheumatoid dysplasia in differential diagnosis of juvenile idiopathic arthritis

**DOI:** 10.1186/1546-0096-11-S2-P253

**Published:** 2013-12-05

**Authors:** E Vrtíková, E Košková, M Genčík, J Sedlákova

**Affiliations:** 1 National Institute of Rheumatic Diseases, Piešťany, Slovakia; 2 Medgene s.r.o., Bratislava, Slovakia

## Introduction

In the differential diagnostics of juvenile idiopathic arthritis (JIA), rare genetic diseases that often mimic chronic polyarthritis, should be considered. In our case report we describe such rare disease - progressive pseudorheumatoid dysplasia (PPD).

## Objectives

A 13 year old female patient started to complain about lower extremity pain at the age of 3 years, refuted to walk, and the walking stereotype has been disrupted. She has been seen at our rheumatology practice at the age of 7 year. Our findings included a baby thickening and rigidity of the proximal interphalangeal joints of the hands, a substantial deficit in hip function, valgus deformities of knees and ankles with painless movement limitation, restriction of the cervical spine function and signs of thoracic kyphosis. Arthrology and X-ray hands examination suggested a polyarticular form of JIA. Blood examination did not show increased inflammatory activity and the platyspondyly shown on the spine X-Ray was also discrepant. Given the atypical clinical representation, we consider Mucopolysaccharidosis which has been excluded by repeated targeted examination. Given the progressive functional deficit, development of erosive changes in the hip joint shown by X-ray and ultrasound examinations, immunosuppressive therapy has been administered for 6 months. After 7 years of disease duration a small effusion in the knee joints appeared. Cytological examination, however, showed non-inflammatory features of the synovial fluid. Based on these findings and the overall course of the disease, we revised our diagnosis to progressive pseudorheumatoid dysplasia (PPD). Molecular genetic testing showed the splicing mutation c.589 of the *WISP3 *gene, which is associated with this autosomal recessive skeletal disease. Subsequently we found consanguinity in parents of our patient. *WISP3 *gene plays a role in the cartilage growth and homeostasis. PPD is spondyloepiphyseal dysplasia characterized by progressive arthropathy, recessive inheritance mode and a prevalence of one in 1 million. First symptoms usually appear before the age of 8 years. Problems typically start as muscle weakness and symmetrical swelling of PIP joints of hands. Thereafter during the course of disease a limitation of joint mobility, articular deformities and pain, accentuation of lumbar lordosis and thoracic kyphosis and/or scoliosis each gradually occur. Affected individuals have normal facial appearance and their intellectual capacity is not affected. PPD diagnosis is based on clinical and X-ray findings. Immunosuppressive therapy is not effective but rehabilitation has an irreplaceable role. The disease does not affect life expectancy; its progressive character often requires joint replacement in the early adult. The disorder may be mistakenly diagnosed as JIA; however X-ray findings (platyspondyly, generalized epiphyseal dysplasia) and absence of inflammatory joint involvement are not typical for JIA.

## Methods

Case report.

## Results

Atypical clinical presentation with laboratory and imaging results should indicate revision of the original diagnosis.

## Conclusion

In rheumatology practice the delay in correct diagnosis and unfortunate indication of long-term not indicated immunosuppressive treatment may not always be avoided.

## Disclosure of interest

None declared.

